# Impact of covid-19 on people living with HIV-1: care and prevention indicators at a local and nationwide level, Santo André, Brazil

**DOI:** 10.11606/s1518-8787.2022056004314

**Published:** 2022-05-18

**Authors:** Elaine Monteiro Matsuda, Isabela Penteriche de Oliveira, Laura Ballesteros Bao, Fernanda Matsuda Manzoni, Norberto Camilo Campos, Beatriz Brajal Varejão, Maristelly Pereira Leal, Vania Barbosa Nascimento, Luís Fernando de Macedo Brígido

**Affiliations:** I Secretaria da Saúde de Santo André Centro Médico de Especialidades – Infectologia Santo André São Paulo Brasil Secretaria da Saúde de Santo André. Centro Médico de Especialidades – Infectologia. Santo André, São Paulo, Brasil; II Instituto Adolfo Lutz Centro de Virologia São Paulo São Paulo Brasil Instituto Adolfo Lutz. Centro de Virologia. São Paulo, São Paulo, Brasil; III Faculdade de Medicina do ABC Pós-Graduação em Saúde Coletiva Santo André São Paulo Brasil Faculdade de Medicina do ABC. Pós-Graduação em Saúde Coletiva. Santo André, São Paulo, Brasil

**Keywords:** HIV Infection, prevention & control, PrEP, Acquired Immunodeficiency Syndrome Virus, COVID-19, Brazil

## Abstract

The world has been dealing with Aids for forty years, covid-19 accentuated societal inequalities and promoted a rupture in care and prevention, including for people living with HIV. We compiled official HIV indicators, analyzed the impact of covid-19 in Brazil, at São Paulo State (SP), and compared it to the municipality of Santo André (in the state of São Paulo), which adopted linkage/retention strategies to mitigate the impact of covid-19. From 2019 to 2020, suppression/adhesion rates remained stable. The number of new treatments decreased both in Brazil (-19.75%) and São Paulo (-16.44%), but not in Santo André, where 80% of new patients started treatment within 30 days from their first TCD4 test (70% in São Paulo and 64% in Brazil). However, PrEP dispensing increased during this period. The distribution of 2,820 HIV self-tests in Santo André lead to only one documented new HIV diagnosis linked to care. Synergistic strategies to swiftly diagnose and connect new cases, ensuring retention as well as rescuing missing patients deserve priority in the fight against HIV, especially in times of covid-19.

## INTRODUCTION

The WHO^1^ declared the coronavirus disease 2019 (covid-19) a pandemic early in 2020. By March 29, 2021, the world had 196,553,009 confirmed cases, including 4,200, 412 deaths. Even though Brazil has 2.7% of the world population, it concentrated 13.3% of all covid-19 cases and 26.9% of all deaths at that time, with more than half a million deaths by July, 2021^2 , 3^.

Meanwhile, 40 years after the first documented Aids cases, its pandemic is far from reaching an end. In 2020 it was estimated that 37,700,000 people were living with HIV (PLWH), 1,500,000 new infections occurred during the year, 680,000 deaths related to Aids, with 73% of PLWH receiving antiretroviral therapy (ART)^4^. All over the world, health services have been disrupted, including those needed to support prevention, diagnosis, and treatment^5 , 6^.

The Brazilian Unified Health System (SUS) ensures access to health and services in a universal, equal, and integral way. Along with free ART for all PLWH and specific policies, Brazil had been a model for Aids response, a leadership that was further shaken in recent years as result of a crisis that originated from cuts and freezes in the budget for health, education, and social assistance^7^. SARS-CoV-2 aggravated this scenario both directly, because of covid-19 disease, and indirectly, by disrupting HIV care^8 , 9^. The covid-19 pandemic has accentuated existing inequalities and made the vulnerable population more vulnerable to hunger, violence, and diseases. The metaphor “we are not in the same boat, but in the same tempestuous sea” expresses the very unequal conditions in coping with covid-19 demands.

### Strategies Adopted at the Santo André Infectious Diseases Outpatient Clinic

Coinciding with the beginning of the first covid-19 wave in Brazil (March 2020), the Santo André infectious diseases outpatient clinic (SA-IDC), the reference in the care of PLWH in the city, started a research project which aimed to test strategies to improve patient linkage/retention, an initiative that was adapted to the restrictions imposed by covid-19. The main strategies to mitigate the impact of covid-19 were: (i) screening at admission, managing users’ needs; ii) maintenance of new patients’ admission (without prior follow-up or transfer) as much as possible, and linking on the same day of admission; iii) maintenance of antiretroviral dispensing; iv) evaluation of complaints and need for laboratory exam collection at ART withdrawal; v) phone monitoring of newly admitted cases with recalls by the nursing staff until viral suppression was achieved; vi) contact with cases with delays in ART withdrawal for over ten days, first by WhatsApp message and, if unanswered, a home visit was made by the social worker; vii) provision of HIV rapid self-test for users seeking them, after discarding post-exposure prophylaxis (PEP) eligibility and; viii) psychological evaluations were maintained, whenever possible, online.

### Official HIV Care and Prevention Indicators

**Figure 1:**
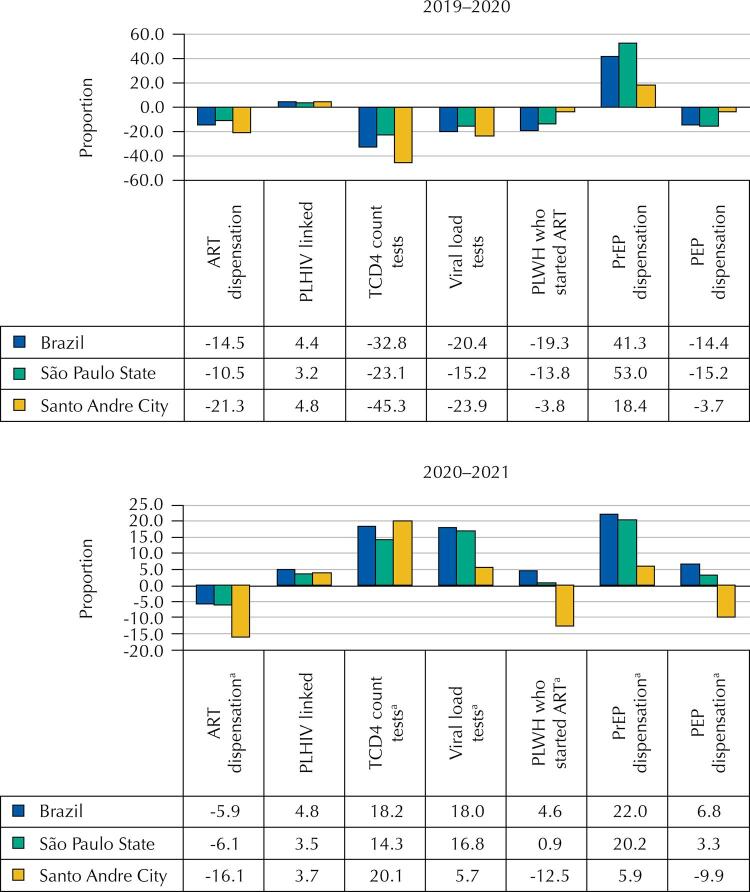
Proportion of variation of covid-19 HIV panel data between 2019–2020 and 2020–2021a.

ART: antiretroviral; PLWH, persons living with HIV; PrEP, pre-exposure prophylaxis; PEP, post-exposure prophylaxis.

^a^ Data for 2021 were estimated from data for the first half of 2021, multiplying them by 2.

Source: Ministério da Saúde (BR) 2020^12^.

We conducted a descriptive study based on data on the Brazilian Ministry of Health official registry of HIV/Aids care and prevention indicators (MS/SVS/Department of Chronic Diseases and Sexually Transmitted Infections) to evaluate whether the strategies adopted in Santo André mitigated the difficulties imposed by the covid-19 pandemic. Information for 2019, 2020, and 2021 (analyzed from figures for its first semester) from Brazil, São Paulo State (SP), and Santo André (SA) were compared^10 , 11^. Number of T CD4 counts, HIV viral load tests, patients starting treatment, and antiretroviral dispensing were obtained from the covid-19 HIV panel^12^. Clinical information^13^ for 2019 and 2020 on linked patients, late, and timely diagnosis, viralsuppression, and adherence to ART were evaluated. Table 1 and 2 summarize the indicators provided by the Ministry of Health. Table 1 shows information from a panel developed to assist and monitor the care provided by states and municipalities concerning the main operational indicators that affect treatment and prevention regarding the HIV epidemic in the years 2019, 2020, and projected for 2021, as well as their variation (2019–2020 and 2020–2021). Figure 1 illustrates the proportional variation of the indicators shown in Table 1 ^12^. Table 2 shows HIV-related clinical indicators for 2019 and 2020^13^.

**Table 1:** HIV care parameters obtained from the covid-19 HIV panel 2019–2021.

		Numbers of ART dispensation	PLWH linked	Numbers of CD4 count tests	Numbers of HIV viral load tests	PLWH that started ART	Numbers of HIV self-tested distribution	Number of PrEP dispensations	Number of PEP dispensations
Brazil	2019	4,844,525	675,786	455,554	919,516	73,030	56,703	50,341	112,613
	2020	4,143,652	705,261	306,009	732,287	58,933	172,606	71,147	96,419
	2021^a^	3,900,442	738,979	361,648	864,430	61,652	93,972	86,834	102,940
	∆2019–2020	-14.5	4.4	-32.8	-20.4	-19.3	204	41.3	-14.4
	∆2020–2021^a^	-5.9	4.8	18.2	18.0	4.6	-45.6	22.0	6.8
São Paulo	2019	1,040,794	163,188	101,954	232,568	13,505	36,772	22,320	41,029
	2020	9,31,576	168,433	78,400	197,128	11,642	92,666	34,144	34,777
	2021^a^	875,110	174,340	89,614	230,226	11,742	54,026	41,048	35,908
	∆2019–2020	-10.5	3.2	-23.1	-15.2	-13.8	152	53.0	-15.2
	∆2020–2021^a^	-6.1	3.5	14.3	16.8	0.9	-41.7	20.2	3.3
Santo André	2019	17,724	2,432	1,249	3,746	183	6	386	733
	2020	13,956	2,549	683	2,852	176	2,820	457	706
	2021^a^	11,716	2,644	820	3,014	154	274	484	636
	∆2019–2020	-21.3	4.8	-45.3	-23.9	-3.8	46,900	18.4	-3.7
	∆2020–2021^a^	-16.1	3.7	20.1	5.7	-12.5	-90.3	5.9	-9.9

ART: antiretroviral; PLWH: persons living with HIV; PrEP: pre-exposure prophylaxis; PEP: postexposure prophylaxis.

^a^ HIV indicators for all the country, São Paulo state and City of Santo André at pre-pandemic (2019) and two pos pandemic years 2020 e 2021, the latter projected from the first semester, and ∆ the variation 2019–2020 and 2020–2021 as percentage of change.

Source: Ministério da Saúde (BR) 2020^12^.

**Table 2:** HIV clinical indicators (2019–2020) in Brazil, São Paulo state and city of Santo André.

		Late diagnosis (%)	ART within 30 days (%)	Linked	Started ART	On ART	Timely start of ART (%)	Viral suppression (%)	Sustained suppression (%)	Sufficient adhesion (%)	Insufficient adhesion (%)	Loss of follow-up (%)
Brazil	2019	26	58	753,316	68,527	633,699	35	88	74	73	18	9
	2020	27	64	757,075	54,995	665,080	35	89	76	78	13	9
	∆2019–2020	1	6	0.50	**-19.75**	4.95	0	1	2	5	-5	0
São Paulo	2019	23	61	179,763	12,820	150,986	38	90	76	75	16	10
	2020	23	70	179,859	10,713	158,608	37	91	78	79	13	8
	∆2019–2020	0	9	0.05	-16.44	5.05	-1	1	2	4	-3	-2
Santo André	2019	27	82	2,714	181	2,381	43	92	78	80	14	6
	2020	23	84	2,724	181	2,449	31	93	80	87	6	7
	∆2019–2020	-4	2	0.37	**0.00**	2.86	-12	1	2	7	-8	1

Data for all the country, only the São Paulo state and only City of Santo André at pre-covid-19 pandemic (2019) and first covid-19 pandemic year (2020), with ∆, variation (percentage).

ART: antiretroviral; Late diagnosis: percentage of individuals with a first count of T CD4 cells record < 200 cells/mL; Treatment within 30 days: Percentage of individuals eligible for ART who started treatment within one month after performed the first count of T CD4 cells; Linked: number of individuals with at least one dispensation, one T CD4 cells or viral load collection; Started ART: number of individuals who started ART; On ART: number of persons living with HIV (PLWH) on ART (at least one medication dispensing, or withdraw, in the last 100 days); Timely start of ART: proportion of individuals starting ART with T CD4 cells > 500 cells/mL; Viral suppression (percentage of viral suppression): all individuals on ART (dispensed in the last 100 days of the year) who performed viral load, whose result was below 50 copies/mL; Sustained suppression: percentage of individuals on ART (withdraw in the last 100 days of the year, for at least two years and who performed at least 02 viral load tests with results below 50 copies/mL after at least 06 months from the beginning of the treatment; Sufficient adhesion: proportion of individuals with more than 80% ART adherence at the end of each year. Insufficient adhesion: proportion of individuals with at least one withdrawal of ARV, but with more than 80% adherence at the end of each year; Loss of follow-up: proportion of individuals without at least one withdrawal at the end of each year.

Source: Ministério da Saúde (BR) 2021^13^.

### Consequences of the covid-19 pandemic: not everything got worse

The response to covid-19 included the cancellation of appointments and the routine collection of tests in all health units in mid-March 2020. The decrease in the number of ART dispensations as treatment shown in Table 1 and Figure 1 , is a result of the increase in the amount of ART dispensed each time from 30 to 90 days, from 8%, 16%, and 6%, in 2019 to 25% in all strata by 2020, representing an increase (17%, 9%, and 19%) in Brazil, São Paulo, and Santo André, respectively. This was an old request that facilitates medication dispensing that should be further expanded. This ART multi-month dispensing policy proved able to contribute both to adherence to HIV treatment as well to a decrease in opportunities for transmission of covid-19^14^, moreover, ART is an fundamental tool to control the HIV epidemic, as the “undetectable=untransmittable” policy shows^15^.

While pre-exposure prophylaxis (PrEP) dispensation increased (41%, 53%, and 18%), post-exposure prophylaxis (PEP) dispensation decreased (14%, 15%, and 4%) in 2020, when compared to 2019, in Brazil, São Paulo, and Santo André, respectively. At least in Santo André, where we had access to the details of PEP use, its decrease was due to the 37% reduction in the demand for accidents with biological material, from 155 in 2019 to 98 in 2020, whereas use for consented sexual exposure increased 6%, from 527 in 2019 to 557 in 2020. The increase coincided with a time when contact should have been restricted due to covid-19. PrEP dispensation increased overall due to efforts of national implementation, which was not so evident in Santo André, due to a reduction in the health care team that lead to the suspension of new PrEP program admissions.

**Figure 2:**
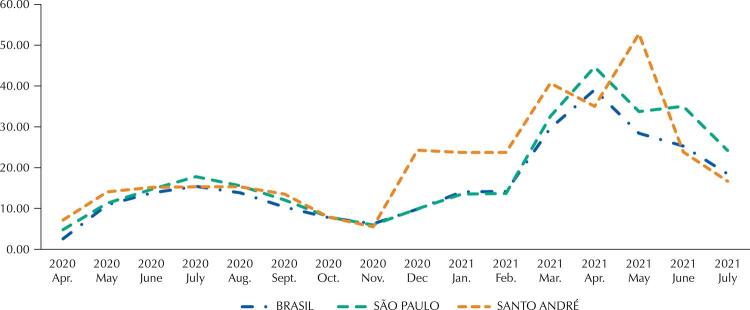
Mortality rate due to covid-19 in Brazil, in São Paulo State, and the municipality of Santo André.

Figure 2 shows mortality rates in Brazil, São Paulo, and Santo André as the result of the first and second infection waves^10 , 11^to contextualize the covid-19 pandemic phases. Covid-19 death rates, shown as deaths per 100,000 inhabitants, were estimated by dividing the number of deaths by the estimated population of 2021 (https://www.ibge.gov.br/) and multiplying the result by 100,000. Thus, we obtained 260 (Brazil), 298 (SP), and 335 (SA) deaths per 100,000 inhabitants. The higher documented death rate in Santo André suggests that the city was proportionally more affected by covid-19, with 12% and 29% more deaths/100,000 inhabitants than the state and country, respectively.

The Santo André public health system prioritizes the diagnosis of HIV infections via HIV rapid tests (RT) performed on SUS health services. The amount of RT performed for diagnosis in 2019, 2020, and in the first half of 2021 was provided by the Santo André health department. Out of 30,910 tests in 2019, 324 (1.1%) were reactive. Out of 28,414 in 2020, 308 (1.1%) were reactive, an 8% decrease in testing. Until June 2021, only 11,997 (113, 0.9% reactive) tests were performed. On the other hand, there was a marked increase in self-tests dispensed, from only six in 2019 to 2,820 in 2020. Those were almost entirely dispensed in the SA-IDC, and 635/2,820 were provided directly in saunas and bars attended by the more vulnerable population. However, among 115 cases admitted without prior follow-up in 2020, only one reported having sought the service due to the reactive result from the self-test obtained free of charge from SUS. 1/2,820, (0.04%) with six additional cases referring a paid self-test acquired in a pharmacy. In the first semester of 2021, only 274 self-tests were dispensed and in-service testing resumed. HIV testing is a cornerstone for prevention and treatment, needed to achieve suppression and break the transmission cycle^15^. Albeit the important increase of self-tests dispensed, our observation may suggest that those who buy a test will more likely use it, whereas an individual who receives a test for free, may not do it properly. This needs further evaluation as the effectiveness of this and other modalities of diagnostic incentive are crucial to inform prevention policy.

New cases linked to the service in the year are estimated by the number of people who started ART. Figure 1 and Table 1 show a different value for people who started ART than what Table 2 shows, as they used different sources. Santo André has 1.5% (n = 2,644) of PLWH linked in the SA-IDC, and 1.6% (n = 723,889) of the estimated state population in 2021. The SA-IDC, in line with SUS guidelines, does not restrict its services exclusively to residents in Santo André, and even during the greater restriction, it maintained the admission of new diagnoses or transferred cases. The maintained number of patients who started ART in 2020, compared to 2021, reflects this policy, whereas we found a documented reduction of 19.8% in Brazil and 16.4% in SP. The Global Fund’s Results Report 2021 confirms the devastating impact of covid-19 on HIV services, including an 11% decline in prevention services and a 22% reduction in HIV treatment initiation^6^.

The proportion of individuals who start ART within a month after collecting their first TCD4 count test (TimeCD4/ART) may be a proxy for how quickly linking to care occurs. Santo André showed faster linking and maintained more than 80% of its patients in 2020, compared with 70% in São Paulo and 64% in Brazil ( Table 2 ). The faster TimeCD4/ART in Santo André reflects an effort started in 2018, which aimed to start treatment within seven days of admission. This policy was not associated to any additional loss to follow-up^16^. With covid-19, we tried to start treatment on the same day, consistent with the fact that in the presence of exposure risk, we prescribe PEP to mitigate the risk of a new infection. Therefore, we could use the same logic for treating quickly to prevent new infections^17^. Worst-case estimates by North American researchers suggest that disruptions in HIV prevention and care during the pandemic may result in a 9% increase in new HIV infections over the next few years^18^. Monitoring laboratory tools suffered a major temporary interruption during the initial response to the pandemic. For a while, there was no evidence for a decrease in sustained suppression. However, longer follow-up data are needed to assume the durability of ART suppression.

Disparities in the social determinants of health and comorbidities likely have a greater influence on the susceptibility of PLWH to covid-19. PLWH bear a disproportionate burden of alcohol, drug use, and mental health disorders, as well as other structural vulnerabilities, which might increase their risk to both viral infections^19^. According to data from 37 countries, HIV infection is independently associated with an increased 30% risk of death among patients hospitalized with suspected or confirmed SARS-CoV-2 infection^6^. These inequalities in confronting covid-19 should not repeat what happened with Aids. Similar imbalances are emerging and repeating mistakes will be unforgivable. Vaccine disparities are the clearest evidence of this gap. The richest countries must act now to ensure that no one is left behind in the coronavirus vaccine rollout^20^.

Although official Brazilian data show stability in retention and viral suppression of PLWH, careful monitoring in the coming years will be necessary, due to the impoverishment the country is experiencing amid a disruption of social public policies^21^, essential for a robust health system. Priorities cannot be neglected, and managers need to use science creatively, adapting the response to local challenges.
